# Epigenetics and Signaling Pathways in Glaucoma

**DOI:** 10.1155/2017/5712341

**Published:** 2017-01-22

**Authors:** Angela C. Gauthier, Ji Liu

**Affiliations:** Department of Ophthalmology and Visual Science, Yale School of Medicine, New Haven, CT 06510, USA

## Abstract

Glaucoma is the most common cause of irreversible blindness worldwide. This neurodegenerative disease becomes more prevalent with aging, but predisposing genetic and environmental factors also contribute to increased risk. Emerging evidence now suggests that epigenetics may also be involved, which provides potential new therapeutic targets. These three factors work through several pathways, including TGF-*β*, MAP kinase, Rho kinase, BDNF, JNK, PI-3/Akt, PTEN, Bcl-2, Caspase, and Calcium-Calpain signaling. Together, these pathways result in the upregulation of proapoptotic gene expression, the downregulation of neuroprotective and prosurvival factors, and the generation of fibrosis at the trabecular meshwork, which may block aqueous humor drainage. Novel therapeutic agents targeting these pathway members have shown preliminary success in animal models and even human trials, demonstrating that they may eventually be used to preserve retinal neurons and vision.

## 1. Introduction

Glaucoma is a group of eye diseases characterized by retinal ganglion cell (RGC) degeneration and optic nerve neuroretinal rim loss. It affects approximately 3.5 percent of the world population aged 40 to 80, and it is most prevalent in those of African descent [1]. The condition is often, although not always, associated with increased intraocular pressure (IOP), which can lead to mechanical impairment, ischemia, oxidative stress, and inflammation of the optic nerve [2]. Patients may be asymptomatic or experience a gradual and subtle loss of peripheral or central vision before more severe visual function loss is noticed. Treatment generally consists of lowering IOP through medications, laser therapy, or surgery, although novel approaches promoting neuroprotection are now incipient [2].

Genetics and environmental influences play key roles in glaucoma development [3]. Studies have found that approximately 16–20% of the risk of primary open angle glaucoma (POAG) is attributable to genetic factors, and first- and second-degree relatives of affected patients are both at risk [4, 5]. The process is governed by a complex inheritance pattern with evidence of gene-gene interaction [6, 7]. Mutations in a variety of genes associated with early-onset glaucoma, including* MYOC*,* CYP1BI*,* FOXC1*,* PITX2*,* PAX6*, and* OPTN*, typically disrupt normal development of the trabecular outflow pathway [7]. Environmental factors that raise IOP, such as high wind instruments, coffee, certain yoga positions, tight neckties, and lifting weights, also seem to contribute to glaucoma [8]. Systemic diseases, such as hypertension or hypotension, hyperlipidemia, diabetes, obstructive sleep apnea, and thyroid disease, are sometimes considered risk factors for glaucoma, but this is controversial [9–16]. Exercise, antioxidants, and a diet rich in omega-6 and omega-3 fat seem to lower IOP and thus decrease risk [8].

Emerging research now implicates epigenetic regulation as an important causal factor for glaucoma. Epigenetics, together with genetics and environmental factors, influences the signaling pathways that are ultimately responsible for disease progression. A better understanding of the mechanisms of glaucoma development is necessary to produce targeted treatment, which may hopefully preserve or even restore vision.

## 2. Epigenetics in Glaucoma

### 2.1. Histone and DNA Modification

Epigenetics is the study of heritable nonencoded genetic changes that turn genes on or off. Examples include activating changes such as histone acetylation and DNA demethylation, repressive changes like histone deacetylation and DNA methylation, and modifications induced by noncoding RNAs, such as MicroRNA and long noncoding RNA (lncRNA). Epigenetic modifications can modulate gene expression and/or alter cellular signaling pathways, which may affect individual susceptibility to various diseases. For example, epigenetic changes have been associated with the development of fibrosis in pulmonary fibrosis and liver disease [17, 18].

Some evidence suggests that the glaucomatous eye is a hypoxic environment [19]. Hypoxia has been shown to induce epigenetic changes in prostate cells, and this effect may extend to other cell types [20]. Hypoxia causes Hypoxia-Inducible Factor 1-*α* (HIF1-*α*) to travel from the cytoplasm to the nucleus, so it can recruit the histone acetyltransferase CBP/p300 coactivator to regulate gene expression [21]. The promotor for HIF1-*α* has a HIF Response Element (HRE) that is methylated when oxygen levels are low [21]. The DNA and chromatin modifications allow HIFs to more easily bind to HREs, stimulating epithelial-to-mesenchymal transition [22, 23]. Epithelial cells change into extracellular matrix-secreting myofibroblasts, which leads to fibrosis. Hypoxia may also cause trabecular meshwork fibrosis in glaucomatous eyes, and this blocks the outflow of aqueous humor, leading to an increased IOP.

Epigenetics regulates retinal development, so disturbances in this regulation may lead to ophthalmologic diseases like glaucoma, optic neuritis, and hereditary RGC degeneration [24]. Histone lysine methyltransferases promote RGC survival by methylating lysines on histones 3 and 4 of the RGC developmental genes,* Ath5* and *β3-nAChR*, thereby increasing transcriptional activity [24]. In addition, acute optic nerve injury has been shown to increase nuclear histone deacetylase 3 activity in dying RGCs [25, 26]. This leads to widespread gene silencing in the apoptotic cells. Interestingly, drugs that inhibit histone deacetylases, such as trichostatin A, induce RGC differentiation and neuritogenesis [27]. This suggests that histone deacetylation may be involved in the pathogenesis of glaucomatous optic neuropathy. Complementarily, increased histone acetylation in the retina was found to be neuroprotective in a mouse model of normotension glaucoma [28]. Mice that fasted every other day were found to have increased retinal histone acetylation, which was accompanied by decreased retinal degeneration, increased visual function, and upregulation of Brain Derived Neurotrophic Factor (BDNF) and catalase [28].

Epigenetic forces that may contribute to glaucoma also manifest in lamina cribrosa cells. A study conducted by McDonnell et al. comparing lamina cribrosa cells from glaucomatous human eyes with those from normal eyes found that glaucomatous eyes had significantly increased global DNA methylation [29]. Genes involved in extracellular matrix production, such as *α*-1 type I collagen and *α*-smooth muscle actin, were upregulated. However, they also found that glaucomatous eyes had more unmethylated DNA in the transforming growth factor-*β* (TGF-*β*) 1 promotor region, causing increased transcription of TGF-*β* [29]. They hypothesized that the generally increased methylation primarily applied to genes besides TGF-*β*, turning them off. This may allow other genes such as TGF-*β* that promote fibrosis to become uninhibited. A similar finding was seen in a mouse model of renal fibrosis. Mice with renal fibrosis had hypermethylation of the* RASAL1* promotor in activated fibroblasts [30]. This permitted more Ras expression, which led to fibroblast proliferation [30].

Epigenetic changes associated with glaucoma may be found in cells beyond the eye as well. A prospective case control study found that patients with POAG have higher levels of DNA methylation in peripheral mononuclear cells than healthy controls [31]. The significance of this finding is still under investigation, but it is clear that glaucoma is associated with epigenetic changes that may be responsible for disease progression.

### 2.2. MicroRNA

Noncoding RNA, such as MicroRNA, may also play a role in glaucoma [32]. MicroRNA is a short piece of RNA that can bind to Messenger RNA, preventing its translation into protein. Glaucoma modulates MicroRNA expression, which may serve as a way to communicate damage from the anterior eye to the posterior eye. For example, trabecular meshwork cells injured by oxidative stress in glaucomatous eyes release MicroRNA-21, MicroRNA-450, MicroRNA-107, and MicroRNA-149 into the aqueous humor [33]. These MicroRNAs travel via the uveoscleral pathway to the peripapillary retina, which may affect the optic nerve [33]. However, other MicroRNAs are downregulated in glaucoma. Rats with increased IOP due to a hypertonic saline eye injection had decreased expression of MicroRNA-181c, MicroRNA-497, MicroRNA-204, Let-7a, MicroRNA-29b, MicroRNA-16, MicroRNA-106b, and MicroRNA-25 in their retinas [34]. Human trabecular cells subjected to oxidative stress also show decreased levels of MicroRNA-483-3p [35]. A microarray analysis study found that glaucoma patients had 11 significantly upregulated and 18 significantly downregulated MicroRNAs in their aqueous humor compared to controls [36]. This alludes to the fact that different MicroRNA families may be protective or damaging in the pathogenesis of glaucoma.

MicroRNA families may protect against glaucoma by reducing fibrosis of the trabecular meshwork. When MicroRNA-483-3p was added to stressed human trabecular meshwork cells, it decreased extracellular matrix production, which lowers fibrosis [35]. MicroRNA-483-3p turns off Smad4, an important player in TGF-*β* pathway-induced fibrosis. In addition, increased expression of MicroRNA-29a in human trabecular meshwork cells decreased the extracellular matrix proteins SPARC, collagen I, collagen IV, and fibronectin [37]. Overexpression of the related MicroRNAb suppressed laminin and fibronectin, achieving a similar outcome [37].

Other MicroRNA families may contribute to glaucoma; blocking these targets may be protective. Mutations in the transcription factor FOXC1 can cause Axenfeld-Rieger syndrome, a disorder of abnormal eye and tooth development that frequently involves glaucoma [38]. MicroRNA-204 decreased the expression of FOXC1 as well as its target genes:* CLOCK*,* PLEKSHG5*,* ITGβ1*, and* MEIS2*, indicating its involvement in the disease [38]. In addition, the inhibition of MicroRNA-100 via viral vector prevented apoptosis in rat ganglion cells subjected to H_2_O_2_ oxidative stress [39]. Blocking this MicroRNA also increased neurite growth and stimulated the prosurvival Akt/ERK pathway. The role of MicroRNAs in glaucoma development is still incomplete, but future studies may clarify their involvement and further investigate the pathways by which they act.

### 2.3. Long Noncoding RNA

LncRNAs are RNA transcripts over 200 nucleotides long that typically do not encompass open reading frames of more than 100 amino acids [40]. They are similar to messenger RNAs in that they are capped and polyadenylated with several exons, but they are also shorter and expressed at decreased levels [40]. Approximately 85% of lncRNAs reside in the nucleus, and the rest are in the cytoplasm [41].

LncRNAs play many roles in cellular maintenance, lineage commitment, and differentiation, and evidence suggests that they are heavily involved in neuronal diversification [40, 41]. They influence gene expression by altering proteins after they have been translated and binding to miRNAs, blocking their ability to affect mRNA [41]. Because lncRNAs are so ubiquitous, mutations in the genes that code for them may lead to a diverse array of diseases. For example, the lncRNA called* ANRIL* (antisense noncoding RNA in the INK4 locus) is a tumor suppressor which is transcribed in the antisense direction of Cyclin Dependent Kinase Inhibitor 2B (*CDKN2B*) [41]. Variants in* ANRIL* have been linked to gliomas, leukemia, melanoma, basal cell carcinoma, breast cancer, ovarian cancer, and pancreatic cancer [42]. In addition, they are associated with several eye conditions, including glaucoma, proliferative vitreoretinopathy, diabetic retinopathy, corneal vascularization, and ocular tumors [41]. A retrospective observational case series analyzing several* ANRIL* single-nucleotide polymorphisms (SNPs) associated with glaucoma found that SNP rs3217992 was linked to an increased cup-to-disc ratio at lower IOPs, indicating a possible connection with normal tension glaucoma [43]. Another* ANRIL *SNP variant, rs4977756, has been named a susceptibility locus for POAG according to a genome-wide association study [44]. Mutations in* CDKN2B* have also been associated with glaucoma, and one such variant (rs1063192) was related to higher levels of* ANRIL* expression [45]. Identification of more* ANRIL* SNPs may eventually allow physicians to screen patients at risk for glaucoma for these alleles, leading to earlier diagnosis and treatment.

## 3. Signaling Pathways in Glaucoma

### 3.1. TGF-*β*

TGF-*β* is a cytokine involved in many signaling cascades that cause differentiation, proliferation, chemotaxis, or fibrosis. There are three isoforms of TGF-*β* (TGF-*β*1, TGF-*β*2, and TGF-*β*3), but TGF-*β*2 has the most relevance to the eye [46]. In healthy eyes, TGF-*β*2 helps mediate corneal healing and scar formation and preserves immune privilege in the anterior segment [46]. However, in glaucomatous eyes, increased TGF-*β*2 activity causes fibrosis by increasing the production and deposition of extracellular matrix proteins in trabecular meshwork cells, thereby blocking the outflow of aqueous humor. Patients with POAG have significantly increased levels of TGF-*β*2 in the aqueous humor compared to people with other types of glaucoma and healthy controls [47, 48]. In fact, treatment of human trabecular meshwork cells with TGF-*β*2 upregulates Plasminogen Activator Inhibitor 1 (PAI-1) gene expression and secretion of fibronectin and PAI-1, which are involved in extracellular matrix production [49]. TGF-*β*2-induced extracellular matrix deposition reduces outflow facility of aqueous humor by 27% in cultured human anterior segments, providing further evidence for its role in the pathogenesis of glaucoma [50].

**Figure 1:**
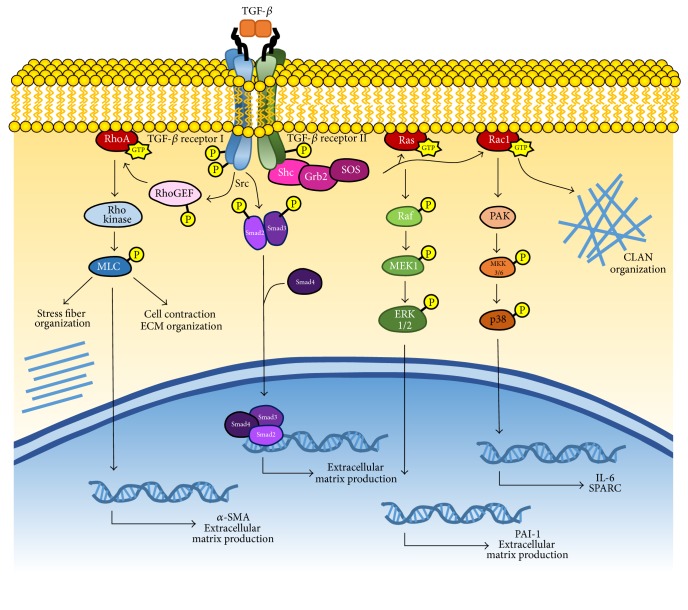
TGF-*β* signaling pathways. TGF-*β* increases extracellular matrix production through the Rho-GTPase/Rho kinase, Smad, and MAP kinase pathways. TGF-*β* binds to TGF-*β* receptors I and II, triggering autophosphorylation. This activates RhoGEF, which attaches a GTP to RhoA. RhoA activates Rho kinase, which leads to the phosphorylation of myosin light chain (MLC). This leads to stress fiber organization, cell contraction, extracellular matrix organization, and the expression of genes for *α*-smooth muscle actin and extracellular matrix production. In the Smad pathway, TGF-*β* binding triggers TGF-*β* receptor I to phosphorylate Smad2 and Smad3, which form a Smad Complex with Smad4. The complex travels to the nucleus, where it helps transcribe genes for extracellular matrix production. TGF-*β* activates the MAP kinase pathway by causing autophosphorylation of the tyrosine residues on TGF-*β* receptor II. This recruits Shc, Grb2, and SOS. SOS activates the GTPases Ras or Rac1. Ras activates Raf, which triggers MEK1 and subsequently ERK 1/2 activation. ERK 1/2 can increase PAI-1 expression in human trabecular meshwork cells, which increases extracellular matrix production. The GTPase Rac1 activates p21-activated kinase (PAK), which activates MAP kinase kinase (MKK) 3/6, which activates p38. This induces expression of Interleukin 6 and SPARC.

TGF-*β* increases extracellular matrix production and remodeling through the canonical Smad pathway as well as noncanonical Mitogen-Activated Protein (MAP) kinase and Rho-GTPase/Rho kinase pathways, which will be discussed in the following sections (Figure 1). In the Smad pathway, TGF-*β* binds to TGF-*β* receptors I and II, causing TGF *β* receptor II to phosphorylate TGF-*β* receptor I [51]. The activated TGF-*β* receptor I then phosphorylates Smad2 and Smad3, which interact with Smad4 to form a Smad Complex. The Smad Complex migrates into the cell nucleus, where it activates the transcription of genes that eventually lead to extracellular matrix production. When TGF-*β*2 is overexpressed in mouse eyes, it causes increased IOP and fibronectin expression in wild-type but not Smad3 knockout mice, demonstrating the importance of the Smad signaling proteins in glaucoma [52].

### 3.2. MAP Kinase

TGF-*β* activates the MAP kinase pathway by first binding to TGF-*β* receptor II, causing autophosphorylation of tyrosine residues (Figure 1). This recruits Src Homology Domain 2 Containing Protein (Shc) and Growth Factor Receptor Binding Protein 2 (Grb2) to bind to the TGF-*β* receptor II [46]. Further binding the Shc-Grb2 complex is Son of Sevenless (SOS), a guanine nucleotide exchange factor (GEF) that activates the GTPases Ras or Rac1. Ras activates Raf, which triggers MAP ERK kinase (MEK) 1 and subsequently extracellular signal-regulated kinase (ERK) 1/2 activation [53]. ERK 1/2 can increase PAI-1 expression in human trabecular meshwork cells, which increases extracellular matrix production [54]. The GTPase Rac1 leads to the activation of p38 MAP kinase pathway, which induces expression of the proinflammatory cytokine Interleukin 6 and Secreted Protein Acidic and Rich in Cysteine (SPARC) in trabecular meshwork cells [55, 56]. SPARC binds to proteins in the extracellular matrix and regulates growth factor efficacy and matrix metalloproteinase expression [57].

The p38 MAP kinase pathway can also be activated when TGF-*β* binds to TGF-*β* receptor I and II, triggering polyubiquitination of TRAF6 at Lys63 [58]. TRAF6 is an E3 ubiquitin ligase, which is physically associated with the TGF-*β* receptors. The polyubiquitination chains hang down and attach to TGF-*β* activated kinase (TAK1), activating it. TAK1 then phosphorylates MAPK kinase 3/6, which then activates the p38 MAPK pathway [59].

### 3.3. Rho Kinase

The Rho family is composed of the Rho, Rac, and Cdc42 subfamilies, which are involved in cell migration, adhesion, proliferation, and actin cytoskeletal organization (Figure 1) [46]. The Rac subfamily has been associated with the development of cross-linked actin network (CLAN) formation, which is seen in trabecular meshwork cells of glaucomatous eyes [60]. Although it is currently unknown how exactly CLANs may cause glaucoma, it has been hypothesized that CLANs can decrease the elasticity of cells, impairing aqueous humor outflow [61]. In addition, trabecular meshwork cells that express a constitutively active form of RhoA, a Rho-GTPase, were found to express increased levels of fibronectin, tenascin C, laminin, *α*-smooth muscle actin, matrix assembly, actin stress fibers, and myosin light-chain phosphorylation, which are associated with the extracellular matrix [62]. These cells were noted to exhibit increased contractile morphology. Rho kinase inhibitors decreased fibronectin and *α*-smooth muscle actin [62]. This suggests that trabecular meshwork rigidity and extracellular matrix production mediated by the Rho pathway may be involved in decreasing aqueous humor outflow, raising IOP.

Whereas Rac activation and subsequent CLAN formation is triggered by the association of Shc, Grb2, and SOS as described above, studies in human choriocarcinoma cells have found that TGF-*β* uses Src-mediated phosphorylation to activate Vav2, a Rho-specific GEF [46, 63]. This pathway eventually leads to the formation of the actin stress fibers that increase cell rigidity [62]. The Rho kinase pathway can also be activated by a variety of factors such as Thromboxane A2, Angiotensin II, Thrombin, Wnt, Endothelin-1, extracellular matrix, and stretch [64]. These factors activate RhoGEF, which activates RhoA, which triggers Rho kinase. Rho kinase initiates many pathways to lead to cell contraction, extracellular matrix organization, *α*-smooth muscle actin expression, and so forth [64].

The Rho kinase pathway has been shown to be a promising target for therapeutics [65]. Rho kinase inhibitors reduce cell rigidity, increasing outflow [66]. Honjo et al. showed that the Rho-associated protein kinase (ROCK) inhibitor Y-27632 increased the outflow of aqueous humor and decreased IOP by 30% after 3 hours in rabbit eyes [67]. Many ROCK inhibitors like netarsudil, RKI-983/SNJ-1656, AR-13324 (Rhopressa®), AR-12286 (Aerie), and AMA0076 (Amakem) are now being tested in clinical trials [66, 68]. Recently, the ROCK inhibitor Ripasudil was approved for the treatment of glaucoma and ocular hypertension in Japan, and it is now being studied for the treatment of diabetic retinopathy [69]. Continued development of ROCK inhibitors will increase the pharmaceutical options available to treat glaucoma and may someday be among the first-line therapies.

### 3.4. BDNF and Other Neurotropic Factors

BDNF is a protein produced by the brain and retina among other organs that supports the growth, differentiation, and survival of neurons. BDNF is especially important for RGC survival [70]. Normally, BDNF and other neurotrophic factors are transported from the brain to the RGCs [71]. However, in glaucoma, the increased IOP blocks axonal transport at the optic nerve head, decreasing neurotrophic levels in the RGCs [72]. The loss of BDNF in these cells contributes to cell death and thus glaucoma through JNK activation and c-Jun phosphorylation, which eventually leads to caspase activation [73, 74]. RGCs try to prevent this outcome by upregulating BDNF production if the optic nerve gets injured. A study done in rats found that after optic nerves were crushed, RGCs expressed elevated BDNF which peaked at 48 hours but declined to baseline levels after two weeks [75]. This effect is neuroprotective, but only temporarily [76]. Nevertheless, preliminary studies in animals show increased RGC survival and even some regeneration after intravitreal neurotrophic factor injection, indicating that it has the potential to eventually become a therapeutic option [77, 78].

### 3.5. c-Jun N-Terminal Kinases (JNKs)

The proapoptotic JNK pathway is initiated by cellular stress, such as ultraviolet radiation, heat shock, and the withdrawal of neurotrophic factors [79]. The aversive stimulus triggers JNK phosphorylation, causing JNK to bind to the N-terminal region of c-Jun [71]. This action phosphorylates c-Jun, a transcription factor for genes that promote apoptosis [80]. It is elevated in the RGCs of rats with induced glaucoma, peaking at one week after the rise in IOP [81]. Optic nerve transection and crush injury also increases c-Jun expression [81–83]. Likewise, humans with glaucoma tend to have elevated levels of phosphorylated JNK in nonglial retina cells [84]. Interestingly, several studies have found that c-Jun activation may also promote survival and regeneration of RGCs, showing that c-Jun may be more versatile than originally thought [85]. Nevertheless, drugs that inhibit JNK tend to guard against RGC loss, providing yet another potential target for neuroprotection [86, 87].

### 3.6. Phosphoinositide-3 Kinase (PI-3 Kinase)/Akt Pathway

The PI-3 kinase/Akt pathway promotes survival and neuroprotection in neurons [88]. Growth factors bind to the membrane-bound tyrosine receptor kinase, which activates PI-3 kinase. PI-3 kinase phosphorylates phosphatidylinositol (4,5)-bisphosphate (PIP_2_) to phosphatidylinositol (3,4,5)-trisphosphate (PIP_3_), which activates Akt. Akt goes on to inhibit the proapoptotic Bcl-2 associated death domain (BAD) proteins, caspases, and the c-Jun pathway [89–91].

Induced IOP elevation in rats has been shown to activate the PI-3 kinase/Akt pathway. Phosphorylated Akt increased on day 1 after translimbal photocoagulation, but it returned to baseline on day 8 [92]. Elevated IOP provoked by episcleral vein cauterization also increased this survival pathway [93]. However, in both of these models, proapoptotic pathways like the MAP kinase pathway, caspase family, Fas ligand, and Fas-Associated Death Domain (FADD) were activated simultaneously, counteracting the prosurvival factors. Ultimately, the proapoptotic protein activation outlasted the neuroprotection pathways, eventually leading to cell death [94].

A variety of neuroprotective drugs that act through different mechanisms have been demonstrated to reduce RGC loss and structural damage through the PI-3 kinase/Akt pathway. These include the prostaglandin analog, Bimatoprost, the fingolimod analog, FTY720, and Vascular Endothelial Growth Factor A [95–97]. Reinforcement of this pathway may be necessary to overcome the longer-lasting proapoptotic factors to achieve enduring neuroprotection.

### 3.7. Phosphatase and Tensin Homologue (PTEN) Pathway

PTEN is a lipid and protein phosphatase that works to inhibit cell growth [98]. It blocks the phosphorylation of PIP_2_ to PIP_3_, preventing activation of the PI-3 kinase pathway and the downstream Akt and mTOR cascades [99].

PTEN has been linked with neurodegeneration as deletion of this gene increases axon regeneration after optic nerve damage [100]. Studies using virus-assisted knockout of PTEN in mice RGCs after crush injury show significant axonal regeneration, especially when the virus is accompanied by another virus encoding ciliary neurotrophic factor and a cyclic adenosine monophosphate analog [101, 102]. However, an even larger effect can be seen in PTEN knockout mice, which are not encumbered by the incomplete gene silencing seen in RNA interference [102]. Mice with a PTEN deletion alone show substantial optic nerve regrowth, but this regeneration only lasts two weeks after the lesion is introduced [103]. However, when PTEN and suppressor of cytokine signaling 3 (SOCS3) were both deleted, the effects lasted well beyond two weeks and there were ten times as many regenerated axons compared to the PTEN-alone group [103]. SOSC3 downregulates the Janus Kinase/Signal Transducer and Activator of Transcription (JAK/STAT) pathway, which is important for cell proliferation. Many of the regenerated axons in these knockout mice have been demonstrated to reach beyond the optic chiasm and even form synapses in the superior colliculus and suprachiasmatic nucleus [103–105]. Furthermore, the suprachiasmatic nucleus showed some neuronal response when the retinal axons were stimulated with light or electricity [105]. However, the responses were not fully functional and the mice did not significantly recover vision [104, 105]. This was because the regrown axons did not have proper myelination, preventing them from conducting adequate action potentials [104]. The addition of voltage-gated potassium channel blockers repaired the conduction deficit, which ultimately enhanced visual function [104].

### 3.8. Bcl-2 Pathway

**Figure 2:**
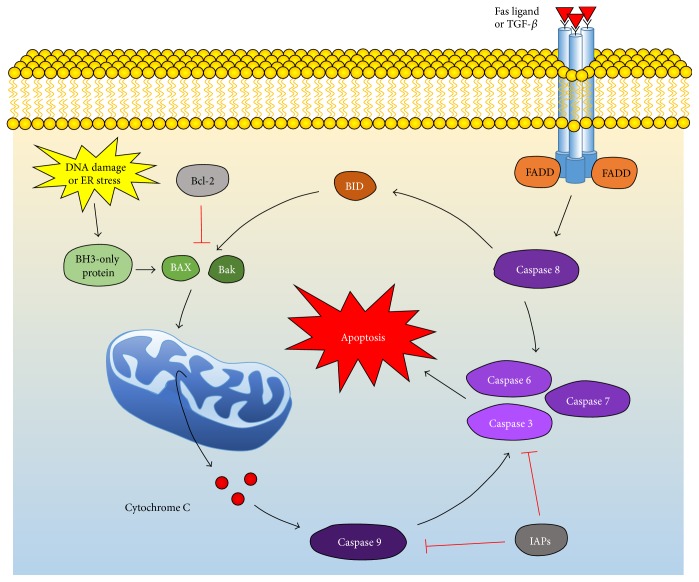
Extrinsic and intrinsic pathways of apoptosis. In the extrinsic pathway, Fas ligand or TGF-*β* binds to its receptor, which is bound by FADD. This activates Caspase 8, which activates the effector Caspases 3, 6, and 7. These caspases all lead to apoptosis. Caspase 8 also activates Bid, a protein that turns on BAX and Bak. The intrinsic pathway also triggers these same proteins by working through BH3-only proteins. BAX and Bak increase the permeability of the mitochondrial membrane, releasing Cytochrome C. This protein activates Caspase 9, which activates the same effector caspases, causing apoptosis. Bcl-2 inhibits this pathway by blocking the activation of BAX and Bak, while IAPs inhibit Caspases 3, 6, 7, and 9.

Bcl-2 is a protein that protects against apoptosis by inhibiting proapoptotic proteins. DNA damage or cellular stress activates BH3-only proteins, which include Bim, Bid, and BAD [71]. These proteins stimulate Bcl-2 associated X protein (BAX) and Bak, which increase mitochondrial membrane permeability and the release of Cytochrome C from the mitochondria (Figure 2). Cytochrome C goes on to activate the caspases that eventually lead to apoptosis. Bcl-2 stops this process by inhibiting BAX and Bak activation [71].

Levkovitch-Verbin et al. showed that optic nerve transection-induced glaucoma and NMDA eye injections increased the expression of BAX and BAD, while downregulating Bcl-2, ultimately leading to cell death [106]. However, overexpression of Bcl-2 leads to neuron preservation, which can even save permanently infarcted brain tissue [107]. In fact, the antibiotic minocycline and the monoamine oxidase B inhibitor rasagiline promote neuroprotection in RGCs by increasing Bcl-2 levels [108, 109]. A number of experimental drugs also protect RGCs through this antioxidant and antiapoptotic pathway, supplying even more targets for intervention [110–112].

### 3.9. Caspases

Caspases are protease enzymes involved in extrinsic and intrinsic apoptotic pathways. In the extrinsic pathway, tumor necrosis factor (TNF) or Fas ligand will bind to the Fas receptor, which is bound to FADD (Figure 2). This activates Caspase 8, which goes on to activate effector Caspases 3, 6, and 7, and these eventually cause apoptosis [113]. In the intrinsic pathway, Cytochrome C released by the mitochondria due to DNA damage or ROS activates Caspase 9. Caspase 9 then activates the same effector caspases, leading to cell death. Apoptosis inhibitor proteins (IAPs) like XIAP, c-IAP, and c-IAP2 inhibit Caspase 3 and Caspase 9, halting this process [113].

Optic nerve transection, crush, and degeneration all result in an increase in Caspase 3 and Caspase 9 activity [114–116]. Caspase 7 knockout mice preserve more RGCs after optic nerve crush than wild-type mice, indicating that blocking caspases may be neuroprotective [117]. Indeed, drugs that inhibit caspases delay RGC death, but they do not help axon regeneration [118]. Neuroprotection is also provided by IAPs, which are upregulated after transection of the optic nerve or induced glaucoma [119]. However, this upregulation only occurs in younger animals and lasts shorter than the concurrently increased expression of proapoptotic genes, causing cells to ultimately die [120].

### 3.10. Calcium-Calpain Pathway

Disruptions in calcium homeostasis occur in many neurodegenerative diseases, including glaucoma [121]. The increased IOP in this disorder intensifies the influx of extracellular calcium into RGCs [122]. Calcium activates calpain, a cysteine protease that cleaves calcineurin [123]. Calcineurin goes on to trigger apoptosis in RGCs via dephosphorylation of BAD and the release of Cytochrome C [124].

The optic nerve crush model has elucidated the role of calcium in axonal degeneration. Axotomy in rat models breaks neuronal membranes and may open voltage-gated calcium channels, allowing the influx of extracellular calcium and the initiation of degeneration [125]. Calcium likely activates the proapoptotic JNK/c-Jun pathway while inhibiting the prosurvival Akt pathway [126]. Calcium ionophores speed up this process [127]. However, the topical addition of calcium channel blockers onto the optic nerve has been shown to block the rise in intracellular calcium, preventing acute superficial axonal destruction [127].

Huang et al. confirmed calpain's role in glaucoma by injecting hypertonic saline into rat eyes and employing immunohistochemistry to look for evidence of calpain activation [123]. They found that the retinas of rats with elevated IOP had a 55 kDa autocatalytic active form of calpain as well as cleaved spectrin and calcineurin, both substrates of calpain. This group also demonstrated that cleaved calpain makes the protease constitutively active, causing it to continuously stimulate the apoptotic pathway in rats with elevated IOP [124]. The inhibition of calpain was shown to decrease RGC death after axonal trauma [126]. When rats were given FK506, a calcineurin inhibitor, there was a marked decrease in BAD dephosphorylation and Cytochrome C release, which in turn promoted survival of RGCs and the optic nerve. Interestingly, optic nerve crush alone did not lead to an increase in calcineurin cleavage [124]. This implies that calcineurin cleavage is not merely triggered by general apoptosis, but rather that it is due to an increase in IOP.

Although the Calcium-Calpain pathway is implicated in axonal degeneration, it is also a necessary factor in growth cone formation that occurs after destruction. New growth cones appear within 24 hours of axonal trauma, and these cones always develop in regions of increased calcium concentration [128, 129]. The proteolytic activity of calpain triggered by increased calcium is necessary to break down proteins needed for regeneration [130]. For example, calpain degrades spectrin, a protein that attaches the intracellular cytoskeleton to the plasma membrane. This may expedite exocytosis which is needed for successful growth cone assembly [125]. The benefits of calcium or calpain inhibition are still unclear as they will inhibit growth cone formation and likely only prevent the acute axonal degeneration that occurs in a small area around the lesion, but further research is needed to investigate this thoroughly [131].

## 4. Conclusions

Glaucoma is a complex disease that can lead to irreversible blindness in many people worldwide. The condition is governed by genetic and environmental factors, and emerging research now suggests a role for epigenetics. These all work through a variety of signaling cascades, including the TGF-*β*, MAP kinase, Rho kinase, BDNF, JNK, PI-3/Akt, PTEN, Bcl-2, Caspase, and Calcium-Calpain pathways. Understanding the molecular players in these pathways is essential for creating new neuroprotective therapeutics that may ultimately help preserve vision.

## References

[1] Tham Y.-C., Li X., Wong T. Y., Quigley H. A., Aung T., Cheng C.-Y. (2014). Global prevalence of glaucoma and projections of glaucoma burden through 2040. A systematic review and meta-analysis. *Ophthalmology*.

[2] Gauthier A. C., Liu J. (2016). Neurodegeneration and neuroprotection in glaucoma. *Yale Journal of Biology and Medicine*.

[3] Wiggs J. L. (2012). The cell and molecular biology of complex forms of glaucoma: updates on genetic, environmental, and epigenetic risk factors. *Investigative Ophthalmology & Visual Science*.

[4] Wolfs R. C. W., Klaver C. C. W., Ramrattan R. S., Van Duijn C. M., Hofman A., De Jong P. T. V. M. (1998). Genetic risk of primary open-angle glaucoma: population-based familial aggregation study. *Archives of Ophthalmology*.

[5] Wang X., Harmon J., Zabrieskie N. (2010). Using the Utah Population Database to assess familial risk of primary open angle glaucoma. *Vision Research*.

[6] Verma S. S., Cooke Bailey J. N., Lucas A. (2016). Epistatic gene-based interaction analyses for glaucoma in eMERGE and NEIGHBOR consortium. *PLOS Genetics*.

[7] Wiggs J. L. (2007). Genetic etiologies of glaucoma. *Archives of Ophthalmology*.

[8] Pasquale L. R., Kang J. H. (2009). Lifestyle, nutrition, and glaucoma. *Journal of Glaucoma*.

[9] Ko F., Boland M. V., Gupta P. (2016). Diabetes, triglyceride levels, and other risk factors for glaucoma in the national health and nutrition examination survey 2005–2008. *Investigative Opthalmology & Visual Science*.

[10] Xu L., Wang H., Wang Y., Jonas J. B. (2007). Intraocular pressure correlated with arterial blood pressure: The Beijing Eye Study. *American Journal of Ophthalmology*.

[11] Zhao D., Cho J., Kim M. H., Guallar E. (2014). The association of blood pressure and primary open-angle glaucoma: a meta-analysis. *American Journal of Ophthalmology*.

[12] Charlson M. E., de Moraes C. G., Link A. (2014). Nocturnal systemic hypotension increases the risk of glaucoma progression. *Ophthalmology*.

[13] Pasquale L. R., Kang J. H., Manson J. E., Willett W. C., Rosner B. A., Hankinson S. E. (2006). Prospective study of type 2 diabetes mellitus and risk of primary open-angle glaucoma in women. *Ophthalmology*.

[14] Girkin C. A., McGwin G., McNeal S. F., Lee P. P., Owsley C. (2004). Hypothyroidism and the development of open-angle glaucoma in a male population. *Ophthalmology*.

[15] Lin C., Hu C., Ho J., Chiu H., Lin H. (2013). Obstructive Sleep Apnea and Increased Risk of Glaucoma. *Ophthalmology*.

[16] Zhao D., Cho J., Kim M. H., Friedman D. S., Guallar E. (2015). Diabetes, fasting glucose, and the risk of glaucoma: a meta-analysis. *Ophthalmology*.

[17] Coward W. R., Watts K., Feghali-Bostwick C. A., Knox A., Pang L. (2009). Defective histone acetylation is responsible for the diminished expression of cyclooxygenase 2 in idiopathic pulmonary fibrosis. *Molecular and Cellular Biology*.

[18] Hardy T., Mann D. A. (2016). Epigenetics in liver disease: from biology to therapeutics. *Gut*.

[19] Tezel G., Wax M. B. (2004). Hypoxia-inducible factor 1*α* in the glaucomatous retina and opticnerve head. *Archives of Ophthalmology*.

[20] Watson J. A., Watson C. J., McCrohan A.-M. (2009). Generation of an epigenetic signature by chronic hypoxia in prostate cells. *Human Molecular Genetics*.

[21] Pennington K., DeAngelis M. (2015). Epigenetic mechanisms of the aging human retina. *Journal of Experimental Neuroscience*.

[22] McDonnell F., O’Brien C., Wallace D. (2014). The role of epigenetics in the fibrotic processes associated with glaucoma. *Journal of Ophthalmology*.

[23] Kimura K., Iwano M., Higgins D. F. (2008). Stable expression of HIF-1*α* in tubular epithelial cells promotes interstitial fibrosis. *American Journal of Physiology—Renal Physiology*.

[24] Rao R. C., Hennig A. K., Malik M. T., Chen D. F., Chen S. (2011). Epigenetic regulation of retinal development and disease. *Journal of Ocular Biology, Diseases, and Informatics*.

[25] Pelzel H. R., Schlamp C. L., Nickells R. W. (2010). Histone H4 deacetylation plays a critical role in early gene silencing during neuronal apoptosis. *BMC Neuroscience*.

[26] He S., Li X., Chan N., Hinton D. R. (2013). Review: epigenetic mechanisms in ocular disease. *Molecular Vision*.

[27] Schwechter B. R., Millet L. E., Levin L. A. (2007). Histone deacetylase inhibition-mediated differentiation of RGC-5 cells and interaction with survival. *Investigative Ophthalmology & Visual Science*.

[28] Guo X., Kimura A., Azuchi Y. (2016). Caloric restriction promotes cell survival in a mouse model of normal tension glaucoma. *Scientific Reports*.

[29] McDonnell F. S., McNally S. A., Clark A. F., O'Brien C. J., Wallace D. M. (2016). Increased global DNA methylation and decreased TGF*β*1 promoter methylation in glaucomatous lamina cribrosa cells. *Journal of Glaucoma*.

[30] Bechtel W., McGoohan S., Zeisberg E. M. (2010). Methylation determines fibroblast activation and fibrogenesis in the kidney. *Nature Medicine*.

[31] Jünemann A., Lenz B., Reulbach U. (2009). Genomic (epigenetic) DNA methylation in patients with open-angle glaucoma. *Acta Ophthalmologica*.

[32] Molasy M., Walczak A., Szaflik J., Szaflik J. P., Majsterek I. (2016). MicroRNAs in glaucoma and neurodegenerative diseases. *Journal of Human Genetics*.

[33] Izzotti A., Ceccaroli C., Longobardi M. G. (2015). Molecular damage in glaucoma: from anterior to posterior eye segment. The MicroRNA role. *MicroRNA*.

[34] Jayaram H., Cepurna W. O., Johnson E. C., Morrison J. C. (2015). MicroRNA expression in the glaucomatous retina. *Investigative Opthalmology & Visual Science*.

[35] Shen W., Han Y., Huang B. (2015). MicroRNA-483-3p inhibits extracellular matrix production by targeting smad4 in human trabecular meshwork cells. *Investigative Opthalmology & Visual Science*.

[36] Tanaka Y., Tsuda S., Kunikata H. (2014). Profiles of extracellular miRNAs in the aqueous humor of glaucoma patients assessed with a microarray system. *Scientific Reports*.

[37] Villarreal G., Oh D., Kang M. H., Rhee D. J. (2011). Coordinated regulation of extracellular matrix synthesis by the microRNA-29 Family in the trabecular meshwork. *Investigative Opthalmology & Visual Science*.

[38] Paylakhi S. H., Moazzeni H., Yazdani S. (2013). FOXC1 in human trabecular meshwork cells is involved in regulatory pathway that includes miR-204, MEIS2, and ITG*β*1. *Experimental Eye Research*.

[39] Kong N., Lu X., Li B. (2014). Downregulation of microRNA-100 protects apoptosis and promotes neuronal growth in retinal ganglion cells. *BMC Molecular Biology*.

[40] Clark B. S., Blackshaw S. (2014). Long non-coding RNA-dependent transcriptional regulation in neuronal development and disease. *Frontiers in Genetics*.

[41] Li F., Wen X., Zhang H., Fan X. (2016). Novel insights into the role of long noncoding RNA in ocular diseases. *International Journal of Molecular Sciences*.

[42] Congrains A., Kamide K., Ohishi M., Rakugi H. (2013). ANRIL: molecular mechanisms and implications in human health. *International Journal of Molecular Sciences*.

[43] Pasquale L. R., Loomis S. J., Kang J. H. (2013). CDKN2B-AS1 genotype–glaucoma feature correlations in primary open-angle glaucoma patients from the United States. *American Journal of Ophthalmology*.

[44] Burdon K. P., MacGregor S., Hewitt A. W. (2011). Genome-wide association study identifies susceptibility loci for open angle glaucoma at TMCO1 and CDKN2B-AS1. *Nature Genetics*.

[45] Ramdas W. D., van Koolwijk L. M., Lemij H. G. (2011). Common genetic variants associated with open-angle glaucoma. *Human Molecular Genetics*.

[46] Pervan C. L. (2016). Smad-independent TGF-*β*2 signaling pathways in human trabecular meshwork cells. *Experimental Eye Research*.

[47] Inatani M., Tanihara H., Katsuta H., Honjo M., Kido N., Honda Y. (2001). Transforming growth factor-*β*_2_ levels in aqueous humor of glaucomatous eyes. *Graefe's Archive for Clinical and Experimental Ophthalmology*.

[48] Ozcan A. A., Ozdemir N., Canataroglu A. (2004). The aqueous levels of TGF-*β*2 in patients with glaucoma. *International Ophthalmology*.

[49] Fleenor D. L., Shepard A. R., Hellberg P. E., Jacobson N., Pang I., Clark A. F. (2006). TGF*β*2-induced changes in human trabecular meshwork: implications for intraocular pressure. *Investigative Opthalmology & Visual Science*.

[50] Gottanka J., Chan D., Eichhorn M., Lutjen-Drecoll E., Ethier C. R. (2004). Effects of TGF-*β*2 in perfused human eyes. *Investigative Ophthalmology & Visual Science*.

[51] Mu Y., Gudey S. K., Landström M. (2012). Non-Smad signaling pathways. *Cell and Tissue Research*.

[52] McDowell C. M., Tebow H. E., Wordinger R. J., Clark A. F. (2013). Smad3 is necessary for transforming growth factor-beta2 induced ocular hypertension in mice. *Experimental Eye Research*.

[53] Zhang Y. E. (2009). Non-Smad pathways in TGF-*β* signaling. *Cell Research*.

[54] Han H., Wecker T., Grehn F., Schlunck G. (2011). Elasticity-dependent modulation of TGF-*β* responses in human trabecular meshwork cells. *Investigative Opthalmology & Visual Science*.

[55] Liton P. B., Li G., Luna C., Gonzalez P., Epstein D. L. (2009). Cross-talk between TGF-*β*1 and IL-6 in human trabecular meshwork cells. *Molecular Vision*.

[56] Kang M. H., Oh D., Kang J., Rhee D. J. (2013). Regulation of SPARC by transforming growth factor *β*2 in human trabecular meshwork. *Investigative Opthalmology & Visual Science*.

[57] Bradshaw A. D., Sage E. H. (2001). SPARC, a matricellular protein that functions in cellular differentiation and tissue response to injury. *Journal of Clinical Investigation*.

[58] Yamashita M., Fatyol K., Jin C., Wang X., Liu Z., Zhang Y. E. (2008). TRAF6 mediates Smad-independent activation of JNK and p38 by TGF-*β*. *Molecular Cell*.

[59] Sorrentino A., Thakur N., Grimsby S. (2008). The type I TGF-*β* receptor engages TRAF6 to activate TAK1 in a receptor kinase-independent manner. *Nature Cell Biology*.

[60] Filla M. S., Schwinn M. K., Sheibani N., Kaufman P. L., Peters D. M. (2009). Regulation of cross-linked actin network (CLAN) formation in human trabecular meshwork (HTM) cells by convergence of distinct *β*1 and *β*3 integrin pathways. *Investigative Opthalmology & Visual Science*.

[61] Clark A. F., Brotchie D., Read A. T. (2005). Dexamethasone alters F-actin architecture and promotes cross-linked actin network formation in human trabecular meshwork tissue. *Cell Motility and the Cytoskeleton*.

[62] Pattabiraman P. P., Rao P. V. (2010). Mechanistic basis of Rho GTPase-induced extracellular matrix synthesis in trabecular meshwork cells. *AJP: Cell Physiology*.

[63] Papadimitriou E., Kardassis D., Moustakas A., Stournaras C. (2011). TGF*β*-induced early activation of the small GTPase RhoA is smad2/3-independent and involves Src and the guanine nucleotide exchange factor Vav2. *Cellular Physiology and Biochemistry*.

[64] Rao P. V., Pattabiraman P. P., Kopczynski C. (2016). Role of the Rho GTPase/Rho kinase signaling pathway in pathogenesis and treatment of glaucoma: bench to bedside research. *Experimental Eye Research*.

[65] Inoue T., Tanihara H. (2013). Rho-associated kinase inhibitors: a novel glaucoma therapy. *Progress in Retinal and Eye Research*.

[66] Prasanna G., Li B., Mogi M., Rice D. S. (2016). Pharmacology of novel intraocular pressure-lowering targets that enhance conventional outflow facility: pitfalls, promises and what lies ahead?. *European Journal of Pharmacology*.

[67] Honjo M., Tanihara H., Inatani M. (2001). Effects of Rho-associated protein kinase inhibitor Y-27632 on intraocular pressure and outflow facility. *Investigative Ophthalmology and Visual Science*.

[68] Sturdivant J. M., Royalty S. M., Lin C. (2016). Discovery of the ROCK inhibitor netarsudil for the treatment of open-angle glaucoma. *Bioorganic & Medicinal Chemistry Letters*.

[69] Garnock-Jones K. P. (2014). Ripasudil: first global approval. *Drugs*.

[70] Johnson J. E., Barde Y.-A., Schwab M., Thoenen H. (1986). Brain-derived neurotrophic factor supports the survival of cultured rat retinal ganglion cells. *Journal of Neuroscience*.

[71] Levkovitch-Verbin H. (2015). Retinal ganglion cell apoptotic pathway in glaucoma: initiating and downstream mechanisms. *Progress in Brain Research*.

[72] Anderson D. R., Hendrickson A. (1974). Effect of intraocular pressure on rapid axoplasmic transport in monkey optic nerve. *Investigative Ophthalmology*.

[73] Almasieh M., Wilson A. M., Morquette B., Cueva Vargas J. L., Di Polo A. (2012). The molecular basis of retinal ganglion cell death in glaucoma. *Progress in Retinal and Eye Research*.

[74] Yuan J., Yankner B. A. (2000). Apoptosis in the nervous system. *Nature*.

[75] Gao H., Qiao X., Hefti F., Hollyfield J. G., Knusel B. (1997). Elevated mRNA expression of brain-derived neurotrophic factor in retinal ganglion cell layer after optic nerve injury. *Investigative Ophthalmology and Visual Science*.

[76] Di Polo A., Aigner L. J., Dunn R. J., Bray G. M., Aguayo A. J. (1998). Prolonged delivery of brain-derived neurotrophic factor by adenovirus-infected Muller cells temporarily rescues injured retinal ganglion cells. *Proceedings of the National Academy of Sciences*.

[77] Nafissi N., Foldvari M. (2016). Neuroprotective therapies in glaucoma: I. Neurotrophic factor delivery. *Wiley Interdisciplinary Reviews: Nanomedicine and Nanobiotechnology*.

[78] Liu Y., Gong Z., Liu L., Sun H. (2010). Combined effect of olfactory ensheathing cell (OEC) transplantation and glial cell line-derived neurotrophic factor (GDNF) intravitreal injection on optic nerve injury in rats. *Molecular Vision*.

[79] Eilers A., Whitfield J., Shah B., Spadoni C., Desmond H., Ham J. (2001). Direct inhibition of c-Jun N-terminal kinase in sympathetic neurones prevents c-jun promoter activation and NGF withdrawal-induced death. *Journal of Neurochemistry*.

[80] Ham J., Eilers A., Whitfield J., Neame S. J., Shah B. (2000). c-Jun and the transcriptional control of neuronal apoptosis. *Biochemical Pharmacology*.

[81] Levkovitch-Verbin H., Quigley H. A., Martin K. R. G. (2005). The transcription factor c-jun is activated in retinal ganglion cells in experimental rat glaucoma. *Experimental Eye Research*.

[82] Isenmann S., Bähr M. (1997). Expression of c-Jun protein in degenerating retinal ganglion cells after optic nerve lesion in the rat. *Experimental Neurology*.

[83] Takeda M., Kato H., Takamiya A., Yoshida A., Kiyama H. (2000). Injury-specific expression of activating transcription factor-3 in retinal ganglion cells and its colocalized expression with phosphorylated c-Jun. *Investigative Ophthalmology and Visual Science*.

[84] Tezel G., Chauhan B. C., LeBlanc R. P., Wax M. B. (2003). Immunohistochemical assessment of the glial mitogen-activated protein kinase activation in glaucoma. *Investigative Ophthalmology and Visual Science*.

[85] Johnson E. C., Guo Y., Cepurna W. O., Morrison J. C. (2009). Neurotrophin roles in retinal ganglion cell survival: lessons from rat glaucoma models. *Experimental Eye Research*.

[86] Sun H., Wang Y., Pang IH. (2011). Protective effect of a JNK inhibitor against retinal ganglion cell loss induced by acute moderate ocular hypertension. *Molecular Vision*.

[87] Tezel G., Yang X., Yang J., Wax M. B. (2004). Role of tumor necrosis factor receptor-1 in the death of retinal ganglion cells following optic nerve crush injury in mice. *Brain Research*.

[88] Dudek H., Datta S. R., Franke T. F. (1997). Regulation of neuronal survival by the serine-threonine protein kinase Akt. *Science*.

[89] Datta S. R., Dudek H., Tao X. (1997). Akt phosphorylation of BAD couples survival signals to the cell-intrinsic death machinery. *Cell*.

[90] Zhou H., Li X., Meinkoth J., Pittman R. N. (2000). Akt regulates cell survival and apoptosis at a postmitochondrial level. *The Journal of Cell Biology*.

[91] Barthwal M. K. (2003). Negative regulation of mixed lineage kinase 3 by protein kinase B/AKT leads to cell survival. *Journal of Biological Chemistry*.

[92] Levkovitch-Verbin H., Harizman N., Dardik R., Nisgav Y., Vander S., Melamed S. (2007). Regulation of cell death and survival pathways in experimental glaucoma. *Experimental Eye Research*.

[93] Kim H. S., Park C. K. (2005). Retinal ganglion cell death is delayed by activation of retinal intrinsic cell survival program. *Brain Research*.

[94] Vander S., Levkovitch-Verbin H. (2012). Regulation of cell death and survival pathways in secondary degeneration of the optic nerve a long-term study. *Current Eye Research*.

[95] Takano N., Tsuruma K., Ohno Y., Shimazawa M., Hara H. (2013). Bimatoprost protects retinal neuronal damage via Akt pathway. *European Journal of Pharmacology*.

[96] You Y., Gupta V. K., Li J. C., Al-Adawy N., Klistorner A., Graham S. L. (2014). FTY720 protects retinal ganglion cells in experimental glaucoma. *Investigative Opthalmology & Visual Science*.

[97] Foxton R. H., Finkelstein A., Vijay S. (2013). VEGF-A is necessary and sufficient for retinal neuroprotection in models of experimental glaucoma. *The American Journal of Pathology*.

[98] Zhu R. L., Cho K. S., Guo C. Y., Chew J., Chen D. F., Yang L. (2013). Intrinsic determinants of optic nerve regeneration. *Chinese Medical Journal*.

[99] Benowitz L. I., He Z., Goldberg J. L. (2017). Reaching the brain: advances in optic nerve regeneration. *Experimental Neurology*.

[100] Koriyama Y., Kamiya M., Arai K., Sugitani K., Ogai K., Kato S. (2014). Nipradilol promotes axon regeneration through S-nitrosylation of PTEN in retinal ganglion cells. *Advances in Experimental Medicine and Biology*.

[101] Park K. K., Liu K., Hu Y. (2008). Promoting axon regeneration in the adult CNS by modulation of the PTEN/mTOR pathway. *Science*.

[102] Yungher B. J., Luo X., Salgueiro Y., Blackmore M. G., Park K. K. (2015). Viral vector-based improvement of optic nerve regeneration: characterization of individual axons' growth patterns and synaptogenesis in a visual target. *Gene Therapy*.

[103] Sun F., Park K. K., Belin S. (2011). Sustained axon regeneration induced by co-deletion of PTEN and SOCS3. *Nature*.

[104] Bei F., Lee H., Liu X. (2016). Restoration of visual function by enhancing conduction in regenerated axons. *Cell*.

[105] Li S., He Q., Wang H. (2015). Injured adult retinal axons with Pten and Socs3 co-deletion reform active synapses with suprachiasmatic neurons. *Neurobiology of Disease*.

[106] Levkovitch-Verbin H., Makarovsky D., Vander S. (2013). Comparison between axonal and retinal ganglion cell gene expression in various optic nerve injuries including glaucoma. *Molecular Vision*.

[107] Martinou J.-C., Dubois-Dauphin M., Staple J. K. (1994). Overexpression of BCL-2 in transgenic mice protects neurons from naturally occurring cell death and experimental ischemia. *Neuron*.

[108] Levkovitch-Verbin H., Vander S., Melamed S. (2011). Rasagiline-induced delay of retinal ganglion cell death in experimental glaucoma in rats. *Journal of Glaucoma*.

[109] Levkovitch-Verbin H., Waserzoog Y., Vander S., Makarovsky D., Ilia P. (2014). Minocycline mechanism of neuroprotection involves the Bcl-2 gene family in optic nerve transection. *International Journal of Neuroscience*.

[110] Wang H., Zhang C., Lu D. (2013). Oligomeric proanthocyanidin protects retinal ganglion cells against oxidative stress-induced apoptosis. *Neural Regeneration Research*.

[111] Cheng H., Ding Y., Yu R., Chen J., Wu C. (2014). Neuroprotection of a novel cyclopeptide C∗HSDGIC∗ from the cyclization of PACAP (1–5) in cellular and rodent models of retinal ganglion cell apoptosis. *PLoS ONE*.

[112] Wang Z., Pan X., Wang D. (2014). Protective effects of protocatechuic acid on retinal ganglion cells from oxidative damage induced by H_2_O_2_. *Neurological Research*.

[113] Marzban H., Del Bigio M. R., Alizadeh J., Ghavami S., Zachariah R. M., Rastegar M. (2015). Cellular commitment in the developing cerebellum. *Frontiers in Cellular Neuroscience*.

[114] Kermer P., Klöcker N., Labes M., Thomsen S., Srinivasan A., Bähr M. (1999). Activation of caspase-3 in axotomized rat retinal ganglion cells in vivo. *FEBS Letters*.

[115] Kermer P., Ankerhold R., Klöcker N., Krajewski S., Reed J. C., Bähr M. (2000). Caspase-9: involvement in secondary death of axotomized rat retinal ganglion cells in vivo. *Molecular Brain Research*.

[116] Levkovitch-Verbin H., Dardik R., Vander S., Melamed S. (2010). Mechanism of retinal ganglion cells death in secondary degeneration of the optic nerve. *Experimental Eye Research*.

[117] Choudhury S., Liu Y., Clark A. F., Pang I.-H. (2015). Caspase-7: a critical mediator of optic nerve injury-induced retinal ganglion cell death. *Molecular Neurodegeneration*.

[118] Vigneswara V., Berry M., Logan A., Ahmed Z. (2012). Pharmacological inhibition of caspase-2 protects axotomised retinal ganglion cells from apoptosis in adult rats. *PLoS ONE*.

[119] Levkovitch-Verbin H., Dardik R., Vander S., Nisgav Y., Kalev-Landoy M., Melamed S. (2006). Experimental glaucoma and optic nerve transection induce simultaneous upregulation of proapoptotic and prosurvival genes. *Investigative Ophthalmology and Visual Science*.

[120] Levkovitch-Verbin H., Vander S., Makarovsky D., Lavinsky F. (2013). Increase in retinal ganglion cells' susceptibility to elevated intraocular pressure and impairment of their endogenous neuroprotective mechanism by age. *Molecular Vision*.

[121] Wojda U., Salinska E., Kuznicki J. (2008). Calcium ions in neuronal degeneration. *IUBMB Life*.

[122] Sappington R. M., Sidorova T., Long D. J., Calkins D. J. (2009). TRPV1: contribution to retinal ganglion cell apoptosis and increased intracellular Ca^2+^ with exposure to hydrostatic pressure. *Investigative Opthalmology & Visual Science*.

[123] Huang W., Fileta J., Rawe I., Qu J., Grosskreutz C. L. (2010). Calpain activation in experimental glaucoma. *Investigative Ophthalmology & Visual Science*.

[124] Huang W., Fileta J. B., Dobberfuhl A. (2005). Calcineurin cleavage is triggered by elevated intraocular pressure, and calcineurin inhibition blocks retinal ganglion cell death in experimental glaucoma. *Proceedings of the National Academy of Sciences*.

[125] Gumy L. F., Tan C. L., Fawcett J. W. (2010). The role of local protein synthesis and degradation in axon regeneration. *Experimental Neurology*.

[126] Ribas V. T., Lingor P. (2016). Calcium channel inhibition-mediated axonal stabilization improves axonal regeneration after optic nerve crush. *Neural Regeneration Research*.

[127] Knoferle J., Koch J. C., Ostendorf T. (2010). Mechanisms of acute axonal degeneration in the optic nerve in vivo. *Proceedings of the National Academy of Sciences*.

[128] Kerschensteiner M., Schwab M. E., Lichtman J. W., Misgeld T. (2005). In vivo imaging of axonal degeneration and regeneration in the injured spinal cord. *Nature Medicine*.

[129] Ziv N. E., Spira M. E. (1997). Localized and transient elevations of intracellular Ca2+ induce the dedifferentiation of axonal segments into growth cones. *Journal of Neuroscience*.

[130] Gitler D., Spira M. E. (1998). Real time imaging of calcium-induced localized proteolytic activity after axotomy and its relation to growth cone formation. *Neuron*.

[131] Lingor P., Koch J. C., Tönges L., Bähr M. (2012). Axonal degeneration as a therapeutic target in the CNS. *Cell and Tissue Research*.

